# Mothercraft and Child-Welfare Centres

**Published:** 1915-10-09

**Authors:** 


### MOTHERCRAFT AND CHILD-WELFARE CENTRES.

### (1) HOW TO MANAGE SCHOOLS FOR MOTHERS.

The instruction of women of the class which should be attracted to schools for mothers is a task not so simple or so easy of accomplishment as many casual observers suppose. The work to be done by those conducting classes of this nature was never more needed than at the present time, and, as we have already pointed out, this work is now aided by grants from the Board of Education. The need for some guidance in the conduct of classes of instruction at schools for mothers has been felt in many quarters, and an instructive Memorandum which has been issued by the Board of Education will undoubtedly be studied with interest by all workers in this comparatively new sphere of activity. The Memorandum itself has been prepared by Dr. Janet M. Campbell, one of the medical officers to the Board, and is obtainable from the usual sources at the modest price of one penny.

It will be acknowledged that many of the disabilities of infancy and childhood are directly traceable to preventible conditions due largely to the mother's lack of knowedge. The subject was discussed in the report for 1913 of the chief medical officer of the Board, and already experience has shown that a good school for mothers is one effective agency for promoting health in infancy, and hence a healthy childhood.

Individual teaching at a school for mothers can scarcely be avoided; special advice in regard to the up-bringing of a particular infant or to the management of a particular home may be extremely valuable; but this method is obviously limited, and some form of collective class teaching on systematic lines must also be provided. At first some individual attention may be the best way of inducing the mother to realise her ignorance and arousing in her a desire to learn, but thereafter every endeavour must be made to maintain as far as possible a regular attendance and a sustained interest.

It is naturally essential that all class instruction be given in the simplest fashion; anything which approaches in character professional or formal lecturing is not likely to bear fruit; it is also necessary to bear in mind that methods must be varied not only to suit the needs of the particular district and the type of women attending a given school, but also with regard to the accommodation available. The scope of the educational facilities of a school for mothers held in a room or hall obtainable only once a week is much more limited than that of a school which has command of the whole of a conveniently sized house.

### Infant Consultations and Mothers' Classes.

In the Memorandum referred to above the general observations are divided into two sections; separate consideration is afforded to (1) teaching given on the same afternoons as the infant consultations are held, and (2) classes held on another day.

With regard to the first condition, there is the obvious advantage that the mothers are already on the school premises, and are generally ready to spend the afternoon at the school. This may be the only occasion in the week, as Dr. Campbell points out, when it is possible to get all the mothers together, and it forms a good opportunity for commencing systematic instruction. This may profitably take the form of a "Health Talk," which should last about a quarter of an hour or twenty minutes; the women cannot be expected to maintain interest for longer than this. After the talk, questions and discussion should be encouraged. A syllabus is useful for the guidance of the teacher, but at any time a matter of special interest at the moment should receive preferential attention; of late, for example, summer diarrhœa. In small classes demonstrations may be supplemented by allowing members to undertake practical work, such as the preparation of fomentations, but with large classes this is not feasible. A sewing class is another form of class teaching which may be held on the consultation afternoons. This should be in full swing by the time the consultations begin; the mothers leave the class one by one to see the doctor and have the baby weighed. Instruction in sewing is organised on educational lines, and includes a blackboard demonstration.

### Mothercraft and Needlework.

In discussing the second series of classes—namely, those held on a day other than that of the consultations—the Memorandum deals with these under three headings: (1) Mothercraft and Hygiene; (2) Needlework; (3) Cookery. Such classes may be attended by mothers who have belonged to the school for some time and have learnt through the informal Health Talks to value instruction.

Mothercraft classes should include lessons on infant care and management, with demonstrations of methods of clothing, washing, and feeding a baby; the making of a simple cradle; the preparation of whey, barley water, and albumin water; together with some elementary teaching on hygiene, first aid, and ventilation. Those who have had any experience in this kind of teaching will cordially agree with the remarks on the advantage of having a blackboard and freely employing it for diagrams and for emphasising words or maxims. It is also very desirable that the number in attendance should not exceed twenty or thirty; larger classes are apt to induce the teacher to give a "lecture" rather than a "talk."

Sewing classes should always be conducted with an educational object. The mothers must be taught to dress children sensibly, and not to overload them with clothes which hamper the chest and leave the rest of the body unprotected from the cold. Then they must learn to make themselves simple garments of a modern pattern, not simply to sew at garments with which they are already too familiar, but they must be taught new ways and about new garments; about the unsuitability of flannelette; they must learn how to cut out each article and its cost, and how to choose the most proper materials. In most cases the women pay the cost price of the material used, and are not allowed to have the completed garment until this has been done. As an encouragement to join the class the mothers at one school in London are presented with a set of baby clothes if they make it themselves.

*(To be continued.)*

